# Verrucous Carcinoma of the Vulva: A Case Report

**DOI:** 10.1155/2013/932712

**Published:** 2013-01-16

**Authors:** Ioannis Boutas, Chrisostomos Sofoudis, Emmanouil Kalampokas, Christos Anastasopoulos, Theodoros Kalampokas, Nikolaos Salakos

**Affiliations:** 2nd Department of Obstetrics and Gynecology and Department of Gynecologic Oncology, “Aretaieion” Athens University Hospital, Thessalonikis Street 59, Kamatero, 13451 Athens, Greece

## Abstract

Verrucous carcinoma of the female genital tract is a rare lesion, primarily affecting postmenopausal women. We present a 78-year-old patient with verrucous carcinoma of the vulva, who was admitted to the “Aretaieion” Athens University Hospital. She had complained of vulvar itching during the last two years without visiting a specialist doctor.

## 1. Introduction

Vulvar cancer is an uncommon lesion of the female genital tract. The different types of vulvar cancer are etiologically heterogeneous. Many conditions have been considered as risk factors for the pathogenesis of this tumor such as HPV infection, smoking, diabetes, and obesity [1, 2]. The use of oral contraceptives and vulvar cancer has not occured primarily among older women; recent studies indicate a significant increase in the occurence of in situ vulvar carcinomas (mainly HPV related), which usually occur in younger women [3].

Verrucous carcinomas (VC) of the vulva represent a distinct entity. It is characterized with slow growing, rarely metastasizing to lymph nodes [4] and is presented as an exophytic-appearing growth that can be locally destructive. The incidence of this type of malignancy is about 1-2% of all gynecological cancers. Verrucous carcinoma has also been found in the oropharynx, perianal, cervix, vagina, penis, scrotum, bladder, and anorectal regions [4–6].

The pathogenesis of VC is not clear yet, but recent studies postulated a putative role for human papilloma virus (HPV) in the etiology [7–9]. Additionally, the role of HPV infection has been confirmed by the detection of viral DNA by CPR [10, 11] in approximately 27% of verrucous carcinomas [12]. 

The treatment of VC is still a matter of discussion. The majority of the scientific community support the fact that a wide local excision should be the best treatment. The previously mentioned therapeutic tactic was selected because of the the lack of spontaneous metastasis. High reoccurrence rates with radiotherapy have been observed [13]. Local application of podophyllin, bleomycin therapy, and cryosurgery are ineffective methods in the treatment of VC [14].

## 2. Case Report

A 78-year-old lady presented to us in 2011 complaining of vulvar itching, pain, and presence of a white lesion during the last two years. From the patients history, it was clear that there were no use of any drug, alcohol, or cigarettes. Five months before the patient presented herself to us, she had undergone a Pap test with vaginal fluid cultivation because of the symptoms described above. Pap test was negative and the vaginal fluid revealed the presence of bacteroides fragilis, treated successfully with metronidazole. One subsequent biopsy of this area during the same period showed histological hyperplastic changes with hyperkeratosis and parakeratosis. 

When the patient came to our center, the general physical examination was normal, but vulvar examination disclosed a white colored induration of the vulva involving both labia majora and the clitoris. The patient underwent into an assiduous imaging and biochemical control. The patient was treated with an extensive excision of the damage. The histological findings confirmed the presence of verrucous carcinoma with tumor-free margins. Following a decision, made by the gynecological oncology team, it seemed appropriate for the patient to undergo radiotherapy. The patient is reviewed every six months thereafter with control imaging and physical and blood examination. The patient until now has not presented any other symptoms.

## 3. Discussion

Verrucous carcinoma of the vulva is a variant of squamous cell carcinoma and is a rare type of vulvar cancer, constituting less than 1% of vulvar cancer overall. The etiology of verrucous carcinoma is not known. However, there have been records showing the presence of HPV genome in the carcinoma tissue [15]. It has been shown that vulvar cancer occurs more frequently among women with primary cancer in another position of the female genital tract and especially the cervix [16]. On the other hand, most vulvar malignancies arise within squamous epithelium [17] and both cervix and vulva are covered by squamous-cell epithelium with a common embryologic origin from the cloacogenic membrane [18].

The diagnosis, also, may be difficult to perform. For this reason, it is convenient to practice large biopsies, in order to avoid misdiagnosis and inadequate treatment.

Verrucous carcinomas exhibit several histological diagnostic criteria such as a “pushing” tumor-dermal interface with minimal stroma between the acanthotic epithelium, minimal nuclear atypia, hyperkeratotic areas on the surface of the tumor with little keratin formation inside the tumor, and diffuse chronic inflammation of the stroma. 

Verrucous carcinomas are locally invasive and rarely metastasize [19]. Usually, it occurs in elderly postmenopausal women, but during the last years an increase incidence of this tumor in younger women (HPV related) has been observed. Women with verrucous carcinoma refer to the specialist because of the presence of a mass in the external genitalia usually itching and sometimes painful. Histologically it presents minimal cellular atypia and very mitotic figures compared with well-differentiated squamous cell carcinoma [20]. Verrucous carcinoma of the vulva may be difficult to treat. In fact, the treatment is still a matter of discussion. Surgery is considered the most effective treatment, but can be associated with local recurrences, especially when the tumor has been inadequately resected [7, 21]. In addition, it is crucial to perform an extensive excision of the ill-defined disease, because of the potential invasion of deep adjacent structures. Verrucous carcinomas are resistant to radiotherapy and sometimes may undergo transformation to squamous cell carcinoma. In some cases, radiation may cause anaplastic transformation, even if this finding is not globally accepted yet [22–25].

The prognosis of VC is relatively good if wide local excision is performed.
